# Round the World

**Published:** 1916-01-15

**Authors:** 


### ROUND THE WORLD.

[The Editor invites Early Information and Contributions Marked "Fellow-Workers."]

### Fellow-Workers and the Roll of Honour.

Lieutenant W. F. Thompson, R.A.M.C., who has died of wounds, received his medical training at St. Bartholomew's Hospital, and obtained the conjoint qualification M.R.C.S., L.R.C.P. in 1912.

Dr. William Everett was among those on the ss. *Persia*, and his name is not among the rescued. Dr. Everett, who was travelling as ship's surgeon, received his medical training at Edinburgh University, and obtained his M.D. degree in 1893. He was at one time senior assistant medical officer to the Kent County Asylum, Chartham.

Dr. Elizabeth Stephens Impey also lost her life in the same disaster. Dr. Impey received her medical training at Birmingham University, and obtained her M.B. degree in 1911. She had held resident appointments at the London Temperance Hospital, the Eye Hospital, Swansea, Birmingham Maternity Hospital, and Birmingham Children's Hospital, and was on her way to Lahore to take charge of the Lady Dufferin Hospital for Women.

Captain R. E. Walker, M.B., R.A.M.C., attached Durham L.I., 2nd Battalion, who is reported wounded in the North of France, was in practice at Peterborough when the war broke out. He took his degree at Edinburgh in 1912, and was for a time senior house surgeon at Edinburgh Royal Infirmary.

Captain J. S. Hall, M.B., R.A.M.C., attached York and Lancaster Regiment, 5th Battalion (T.F.), who has been wounded, obtained his medical training at Glasgow University, becoming M.B., Ch.B. in 1911.

Colonel the Hon J. L. Beeston, Army Medical Corps, Australian Imperial Force, recently had the honour of being received by the King, when His Majesty invested him with the insignia of a Companion of the Order of St. Michael and St. George.

Captain J. V. L. Grant, M.B., R.A.M.C., attached R.H.A., Warwick Battalion (T.F.), is reported wounded. He received his medical training at Edinburgh University, and obtained his medical and surgical degrees in 1911. Captain Grant was formerly house surgeon at the Victoria Hospital, Burnley. When war broke out he was in practice in Edinburgh.

Captain J. E. Brydon, M.B., R.A.M.C. (T.F.), attached R.F.A., Northumbrian Division Ammunition Column, who is reported wounded, was formerly house surgeon at the Darlington Hospital, and prior to that held a resident post at the Bristol Children's Hospital. He received his medical training at Edinburgh University, taking his M.B., Ch.B. in 1908.

Colonel C. F. Pollock, R.A.M.C., whose death recently occurred at Dumfries, at the age of seventy-one, was at one time ophthalmic surgeon to the Royal Hospital for Sick Children, Glasgow, and surgeon for diseases of the eye at the Glasgow Central Dispensary. Some thirty years ago he published a treatise on "Normal and Pathological Histology of the Human Eye and Eyelids," and later a treatise on "Leprosy as a Cause of Blindness." He was educated at Glasgow, Tübingen, and Vienna; he obtained the M.D. degree of Glasgow University in 1882, and became F.R.C.S. Edinburgh in 1884.

